# Why are bleeding trauma patients still dying? Towards a systems hypothesis of trauma

**DOI:** 10.3389/fphys.2022.990903

**Published:** 2022-09-06

**Authors:** Geoffrey P. Dobson, Jodie L. Morris, Hayley L. Letson

**Affiliations:** Heart and Trauma Research Laboratory, College of Medicine and Dentistry, James Cook University, Townsville, QLD, Australia

**Keywords:** hemorrhagic shock, brain, trauma, immune, inflammation, coagulopathy, glycocalyx, ALM

## Abstract

Over the years, many explanations have been put forward to explain early and late deaths following hemorrhagic trauma. Most include single-event, sequential contributions from sympathetic hyperactivity, endotheliopathy, trauma-induced coagulopathy (TIC), hyperinflammation, immune dysfunction, ATP deficit and multiple organ failure (MOF). We view early and late deaths as a systems failure, not as a series of manifestations that occur over time. The traditional approach appears to be a by-product of last century’s highly reductionist, single-nodal thinking, which also extends to patient management, drug treatment and drug design. Current practices appear to focus more on alleviating symptoms rather than addressing the underlying problem. In this review, we discuss the importance of the system, and focus on the brain’s “privilege” status to control secondary injury processes. Loss of status from blood brain barrier damage may be responsible for poor outcomes. We present a unified Systems Hypothesis Of Trauma (SHOT) which involves: 1) CNS-cardiovascular coupling, 2) Endothelial-glycocalyx health, and 3) Mitochondrial integrity. If central control of cardiovascular coupling is maintained, we hypothesize that the endothelium will be protected, mitochondrial energetics will be maintained, and immune dysregulation, inflammation, TIC and MOF will be minimized. Another overlooked contributor to early and late deaths following hemorrhagic trauma is from the trauma of emergent surgery itself. This adds further stress to central control of secondary injury processes. New point-of-care drug therapies are required to switch the body’s genomic and proteomic programs from an injury phenotype to a survival phenotype. Currently, no drug therapy exists that targets the whole system following major trauma.

## Introduction

Their injuries have been fixed surgically, and their incision sites closed. But, some hours or days later, up to 25% of them will still die.

Brohi and colleagues (Brohi et al., 2019) p709

## A global challenge in trauma care

**FIGURE 1:**
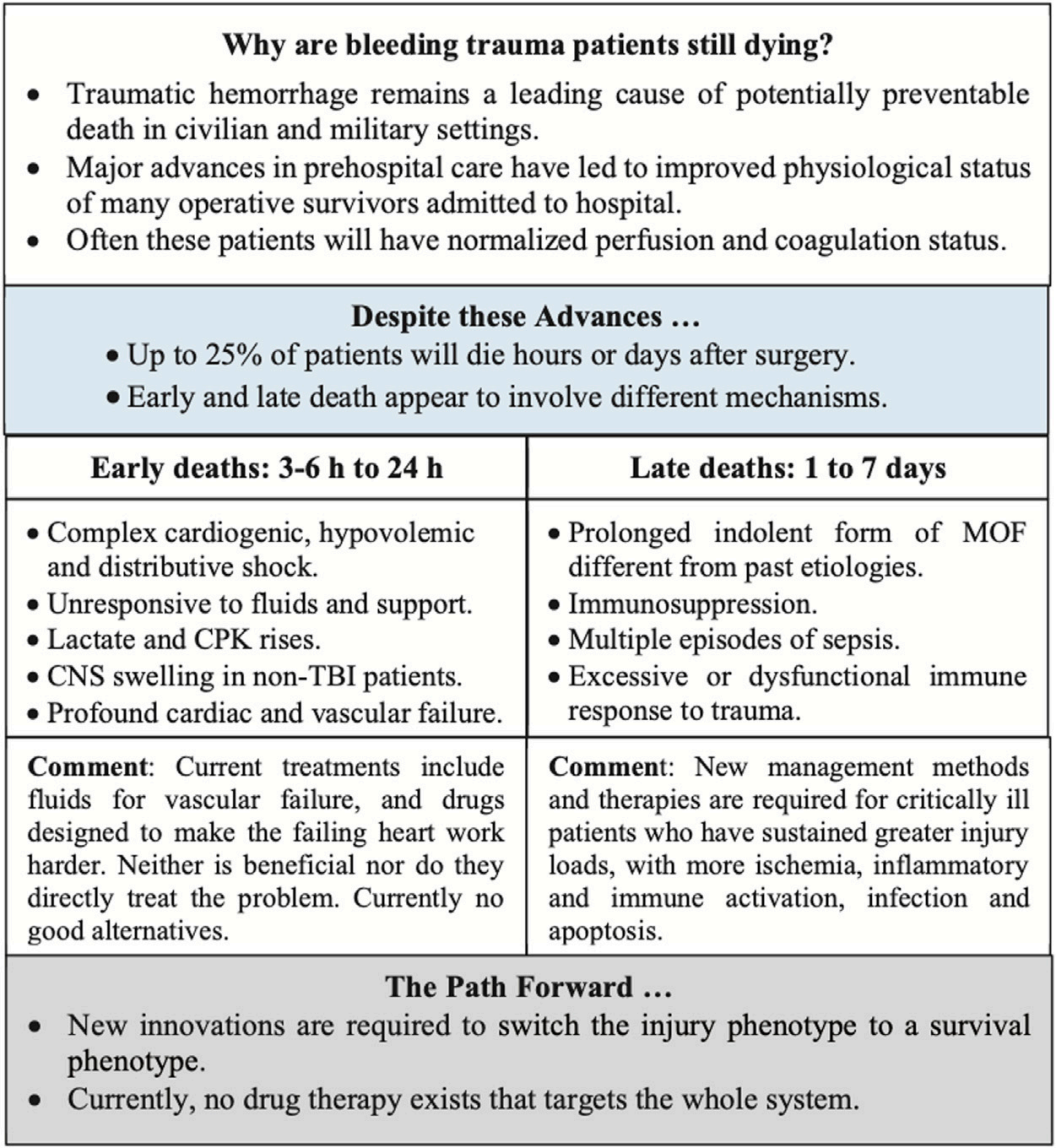
Summary of the characteristics of early and late deaths after traumatic hemorrhage modified after Brohi and colleagues (Brohi et al., 2019). Patients who died at the point-of-injury or in the first few hours upon arrival to hospital have not been included. CNS, central nervous system; CPK, creatine phosphokinase; MOF, multiple organ failure; TBI, traumatic brain injury.

Traumatic hemorrhage remains a leading cause of potentially preventable death in civilian and military environments (Eastridge et al., 2012; Spinella and Cap, 2017; Dobson et al., 2020; Dobson and Letson, 2020; Qasim et al., 2022). Despite advances in trauma care and treatment guidelines, injury and associated morbidity and mortality rates continue to rise (Bedard et al., 2020). In 2019, Brohi and colleagues set a challenge to trauma surgeons, clinicians and scientists to explain why up to 25% of trauma patients, often admitted to hospital with normalized perfusion and coagulation status, are still dying despite receiving the best medical care (Brohi et al., 2019). The first group of early deaths occur ∼3–6 to 24 h after injury and appear to be associated with profound cardiac and vascular failure. The second group of late deaths occur at ∼1 to 7 days and appear to be associated with an indolent form of MOF, immunosuppression and sepsis, referred to as persistent inflammation, immunosuppression and catabolism syndrome (PIICS) (Figure 1) (Brohi et al., 2019). Brohi and colleagues further conceded that: “We have little understanding of why this fulminant cardiac and vascular failure occurs and how to prevent it” or “how to better manage prolonged ischemia, cardiogenic shock, persistent multiple organ dysfunction and immunoparesis” (Brohi et al., 2019). This is a major challenge for trauma research globally.

In this review, we tackle this complex question and argue that a missing piece of the puzzle is the lack of a testable, unified hypothesis of trauma, and no effective point-of-care drug therapies to treat it. We view early and late deaths as systems failures, not as a series of single-event manifestations that occur over time. Breaks in the system appear to occur early from the brain losing its ‘privilege’ status over the rest of the body. After briefly presenting the concept of homeostasis and system design tolerances, we discuss possible breaches to central command control from the surge of damage signals from the primary injury and trauma of emergent surgery itself, and then present a Systems Hypothesis Of Trauma (SHOT) to help explain early and late deaths.

## Homeostasis, design tolerances and physiological reserve

The coordinated physiological processes which maintain most of the steady states in the organism are so complex and so peculiar to living beings - involving, as they may, the brain and nerves, the heart, lungs, kidneys and spleen, all working cooperatively - that I have suggested a special designation for these states, *homeostasis*.

Walter B. Cannon (Cannon, 1932) p20-24

Living systems are steady-states requiring a continual flow of matter, energy and exchange with the environment (Dobson, 2004; Dobson and Letson, 2016). Each system has evolved a state of constancy with operational limits or tolerances, which Walter Cannon termed homeostasis (see quote above). If a trauma (or infection) exceeds the system’s design tolerances, a CNS-driven stress response activates sympathetic outflows with immune activation, inflammation and hemostasis in an attempt to quickly restore homeostasis (Dobson, 2020a; Dobson et al., 2020). If a trauma patient requires emergent surgery, the body is further stressed by the next surge of damage signals from the trauma of surgery itself, despite a successful operation (Dobson, 2020a). We return to this point later. The patient’s response will depend upon type and severity of trauma, extent of hemorrhage, prehospital factors, hospital care proficiencies, time to surgery, and the patient’s physiological reserve, which itself depends upon age, sex and other genetic and non-genetic factors (Bedard et al., 2020; Dobson et al., 2022a).

## Traumatic hemorrhage as a systems failure: Beyond reductionism

After physiology has taken Humpty Dumpty apart, it is difficult perhaps (even unfashionable) to put him back together again. Consequently, traditional analytical approaches like those in physiology can be positively misleading when applied to problems involving the performance of intact organism.

George Bartholomew (1919–2006) (Bartholomew, 1986) p327

Bartholomew’s point is important. For decades, scientists have been trained to reduce a complex living system into its simpler parts, which makes it more amenable to study. A staggering amount of new and valuable information and insight have been generated from this approach (Dobson, 2020b). However, in recent times it appears much less fashionable to put highly mechanistic humpty “back together again” (Dobson et al., 2020; Dobson et al., 2022b). This gap or disconnect can be traced to the molecular revolution of the 20th century, which focused more on mechanisms at the expense of the intact system (Dobson, 2005; Strange, 2005). In the 1960s, Nobel Laureate Sir Francis Crick embodied the reductionist position when he wrote “The ultimate aim of the modern movement in biology is to explain all biology in terms of physics and chemistry” (Crick, 1966; Van Regenmortel, 2004). At the turn of the century, Bloom referred to our current understanding from this approach as “naïve reductionism”, a belief that reductionism alone is incomplete (Bloom, 2001; Strange, 2005). In other words, the enormously rich mother lode of genetic information amassed since the discovery of the double helix in 1953 needs to be placed in context of the phenome. Anticipating the problems, the United States Food and Drug Administration (FDA) in 2004 recommended that: “strengthening and rebuilding the disciplines of physiology, pharmacology and clinical pharmacology, will be necessary to provide the capacity to develop and evaluate new biomarkers and bridge across animal and human studies” (US Food and Drug Administration (FDA), 2004). This FDA Critical Path Initiative remains an ongoing challenge and it highlights the ongoing problem of reductionism in basic science and its relevance to humans.

Students of medicine and biomedicine need to appreciate more that probing the underlying mechanisms of how drugs affect cells or tissue culture is only one tiny step toward understanding how they will behave inside a living organism (Dobson et al., 2019). The price we pay in the current ‘omics’ era is that as science drills deeper and deeper into life’s hidden secrets, we have less and less knowledge on how the parts make up the whole (Dobson, 2005). Indeed, this thinking has influenced the way we study, diagnose, treat, and prevent diseases (Ahn et al., 2006). The current practice of identifying and treating one defect at a time, and so on down the line, often leads to what US surgeon William C. Shoemaker considered: “an uncoordinated and sometimes contradictory therapeutic outcome” (Shoemaker and Beez, 2010). Over many decades, the reductionist practice of naive ‘phenotypic characterization’ has spilled over to the pharmaceutical industry and drug design, which has almost exclusively focused on single-nodal targets. Trauma systems and clinical practice, and research more widely, have also fallen prey to this mind-set. There is an urgent need in the future to incorporate phenotypic and ‘omics’ data into health intervention research. A better understanding on why bleeding trauma patients are still dying may come from rethinking the problem from a systems perspective.

## The forgotten legacy of George Crile: A focus on central control

Traumatic impulses are not excluded by ether anaesthesia from that part of the brain that is apparently asleep.

George W. Crile (Crile, 1913) p7

According to Crile (1864–1943) the brain is never asleep after major trauma or surgery (Dobson, 2020a; Dobson et al., 2021a). Crile was a pioneer of neurosurgery, surgical shock, perioperative protection and regional anesthesia (Katz, 1993; Loop and Crile, 1993; Dobson, 2020a; Dobson et al., 2021a). As an intern, he witnessed one of his friends die from shock after amputation of both legs, and wrote, “I was overwhelmed by my lack of understanding of what was happening and baffled over the inefficiency of treatment” (Crile and Crile, 1936). In subsequent animal studies to educate himself, Crile noticed that after the first incision there was a reflex fluctuation in blood pressure (BP), but not after subsequent incisions (Crile, 1911; Nathoo et al., 2005). *He concluded that the anesthetised brain was wide awake to traumatic impulses from the first incision.*

In an attempt to better protect the brain, Crile proposed “anoci-anesthesia”, which comprised regional use of novocaine (procaine) to block the ‘nerve trunks’, and nitrous oxide gas for general anesthesia, and/or other combinations with morphine and cocaine (Crile, 1911; Crile, 1913; Katz, 1993; Berthelsen, 2015). Anoci was a term he used to describe any factor that protected, preserved, restored or maintained the system (Crile, 1913), which has some similarities to Cannon’s principles of restorative homeostasis (see above). Crile’s goal was to prevent the impulses of stress and pain from reaching the brain, which he believed led to a lowering of the patient’s physiological reserve and “exhaustion of the vasomotor centre” and poor surgical outcomes (Crile, 1913; Loop and Crile, 1993). Crile’s method was a ‘total patient’ systems approach (Dobson, 2020a; Dobson et al., 2021a).

Today, along with the side-effects of general anesthesia, we know that the ‘traumatic impulses’ come from at least two sources following major trauma or surgery. First, as Crile noted, were signals from multiple pain reflex arcs from peripheral and visceral injured sites to higher brain centers (Dobson, 2020a), and second, were alarm signals from injured, stressed or dying cells (Bianchi, 2007; Venereau et al., 2015; Roh and Sohn, 2018). With respect to reflex arcs, an interesting 2008 study of Wennervirta and colleagues evaluated the nociception/antinociception balance during major surgery from finger photoplethysmographic waveform amplitudes and pulse-to-pulse intervals (Wennervirta et al., 2008). They found that their computed surgical stress index (SSI) was lower in patients with plexus block covering the sites of nociceptive stimuli, and that SSI performed better than heart rate, BP, or response entropy (Wennervirta et al., 2008). This is an area of clinical importance that may improve patient outcomes.

**FIGURE 2:**
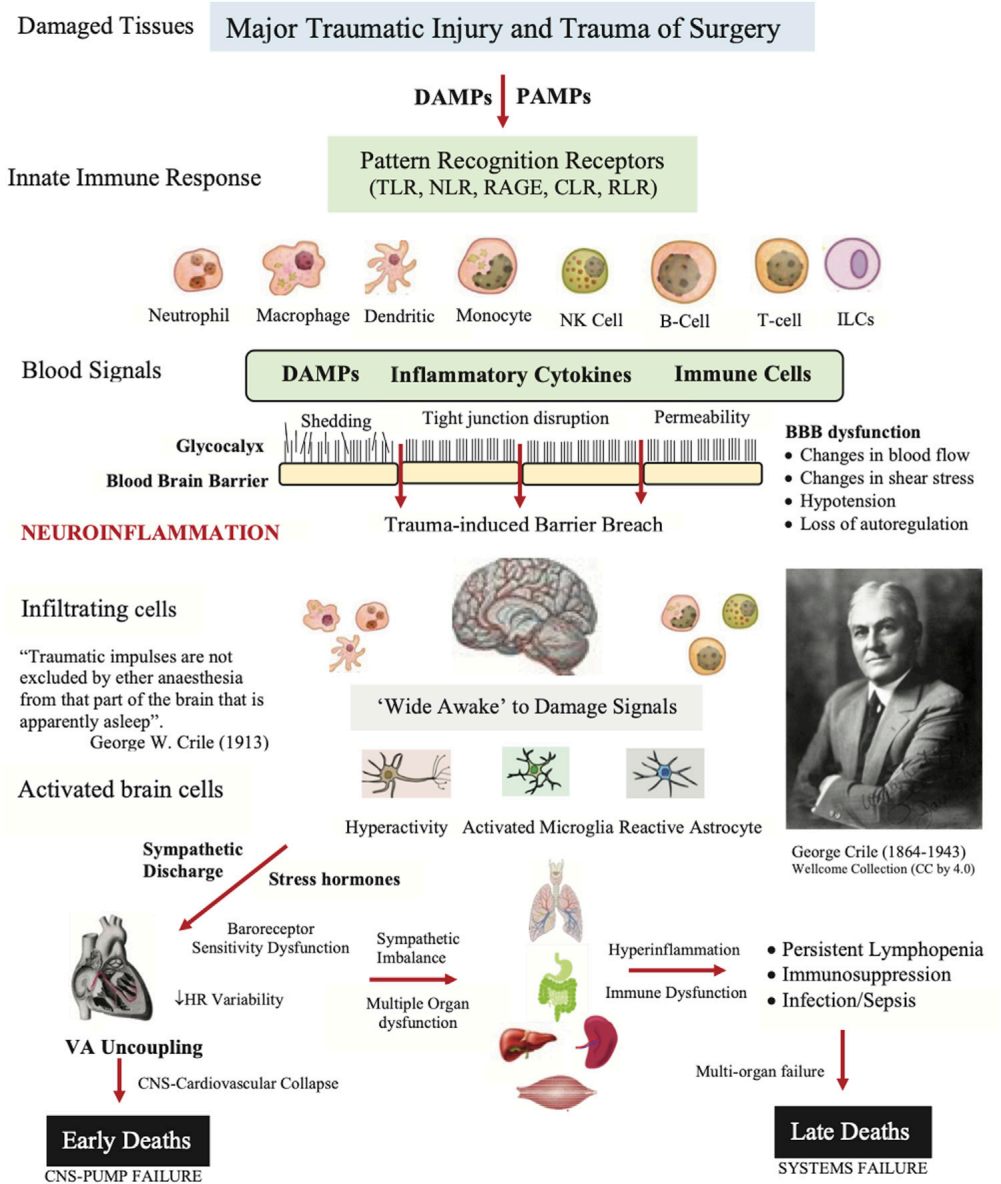
Role of damage associated molecular patterns (DAMPs), pathogen associated molecular patterns (PAMPs), neural signals, and other immune-modifying triggers on disrupting the blood brain barrier (BBB) and causing secondary injury following trauma. The innate immune response involves activation of pattern recognition receptors (PRRs) on resident and blood-borne immune cells including Toll-like receptors (TLRs), C-type lectin receptors (CLRs), nucleotide-binding oligomerization domain (NOD)-like receptors (NLRs), retinoic-acid-inducible gene-I (RIG-I)-like receptors (RLRs), and receptor for advanced glycation end products (RAGE). The brain and immune cells orchestrate the right response by activating neural circuits, and releasing hormones, cytokines and other immuno-inflammatory factors. If dysregulation is persistent, the response can overwhelm the system and lead to multiple organ dysfunction syndrome (MODS) and late deaths. The patient’s response to the trauma of surgery may also contribute to a systems failure (see text). ARDS, acute respiratory distress syndrome; HR, heart rate; ILC, innate lymphoid cell; NK, natural killer cell.

The second source of alarm signals during major surgery are called damage-associated molecular patterns (DAMPs) (Matzinger, 2012), which are derived from the cell membrane, cytosolic, cytoskeleton, nuclear mitochondrial, endothelial and/or blood components that flood into the circulation and can enter the brain following trauma (Figure 2) (Bianchi, 2007; Roh and Sohn, 2018). Early damage markers include high mobility group box protein 1 (HMGB1), mitochondrial DNA (mtDNA), S100, cell fragments, and many other molecules from injured or dying cells as well as proteoglycans and glycoproteins from endothelial-glycocalyx shedding (Muire et al., 2021) (see below). In the case of infection, the damage signals are termed pathogen associated molecular patterns (PAMPs) and other immune-modifying triggers (Venereau et al., 2015). Together, they activate the body’s early immune and inflammatory systems to dial in the right response to repair and restore function (Dobson et al., 2021b; Dobson et al., 2022b). Importantly, DAMPs and PAMPs are not mutually exclusive and may share co-receptors and accessory molecules (Piccinini and Midwood, 2010)*.* Following on from Crile’s and Cannon’s ideas, we argue below that the ‘awake’ brain, with or without anesthesia, is a major player in the failure of the system to restore its homeostatic balance following traumatic hemorrhage (Figure 2).

## Systems Hypothesis Of Trauma

It should be remembered always that the patient who has been in shock and resuscitated, and then operated upon, is in a precarious state. His nervous system has been disturbed not only by the original trauma, but also by the low nutrient flow of blood, and by the surgical procedures incidental to operation.

Walter B. Cannon Quoted from Traumatic Shock (Cannon, 1923) p192

**FIGURE 3:**
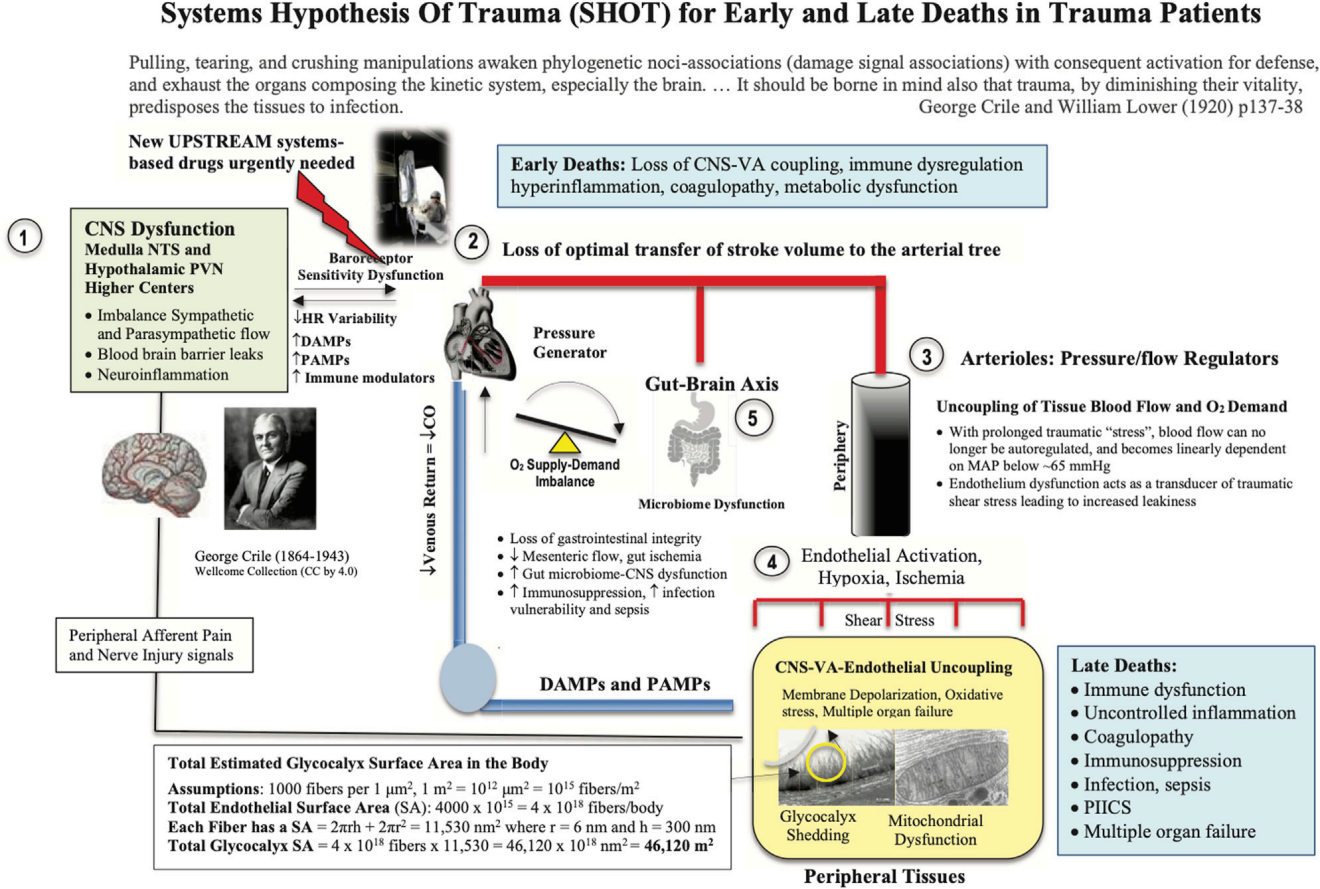
Systems Hypothesis Of Trauma (SHOT) showing key sites of uncoupling involving the: 1) brain, 2) heart, 3) vasculature, 4) endothelial-glycocalyx-mitochondrial functional unit, and 5) gut. Our hypothesis is that if central and local control of cardiac output and ventriculo-arterial (VA) coupling are improved, shear stress will be minimized, blood flow will be optimized, endothelial and microvascular function will be improved, and tissue O_2_ delivery will be maintained. The injury phenotype is driven by sympathetic discharge, an increase in stress hormone levels, immune dysregulation and inflammation along with loss of baroreceptor sensitivity and reduced heart rate variability (Dunser and Hasibeder, 2009; Huston and Tracey, 2011; Matteoli and Boeckxstaens, 2013). Loss of normal central control may be attributed to the disruption to the BBB. New drugs are required to protect the brain and support VA coupling to maintain tissue oxygenation (Dobson, 2014). Details on the SA of the endothelial glycocalyx can be found in Dobson et al., (Dobson et al., 2022b). BBB, blood brain barrier; ATP, adenosine triphosphate; CNS, central nervous system; CO, cardiac output; Cytaa_3_, cytochrome aa3; HIF-1, hypoxia inducible factor 1; HR, heart rate; MAP, mean arterial pressure; NFκB, nuclear factor kappa-light-chain enhancer of activated B cells; NO, nitric oxide; NTS, nucleus tractus solitarius; PIICS, Persistent Inflammation, Immunosuppression and Catabolism Syndrome; PVN, paraventricular nucleus of the hypothalamus.

SHOT was originally formulated in 2015 as an ‘uncoupling’ hypothesis (Dobson, 2015), and since then has undergone a number of iterations to include hemorrhagic shock (Figure 3) (Dobson and Letson, 2016; Dobson, 2020a; Dobson et al., 2020; Dobson and Letson, 2020). SHOT has three pillars of protection.

1) CNS-Cardiovascular Coupling (Systems Controller)
2) Endothelial Glycocalyx Health (Systems Integrator)
3) Mitochondrial Integrity (Systems Regulator)

## First pillar: CNS-cardiovascular coupling

Cannon’s (and Crile’s) insight into the CNS control of traumatic injury cannot be overstated (Dobson, 2015; Dobson et al., 2020). *SHOT predicts that if the CNS stress response can be suppressed early, the downstream secondary injury processes can be attenuated* (Dobson, 2020a). Targeting the hypothalamic–pituitary–adrenal (HPA) axis and the nucleus tractus solitarius (NTS) (regulator of sympathetic and parasympathetic outflows), will maintain the coupling of cardiovascular function to deliver sufficient oxygen to tissue mitochondria (Dobson et al., 2022b; Letson et al., 2022). Shifting autonomic balance from sympathetic ‘hyperdrive’ towards parasympathetic dominance would rebalance the system to reduce immune dysregulation and inflammation and improve end-organ functions (Tracey, 2009; Huston and Tracey, 2011; Lombardi and Stein, 2011; Reyes del Paso et al., 2013; Olshansky, 2016; Sykora et al., 2016; Johansson et al., 2017a; Dobson and Letson, 2020; Letson et al., 2022).

**FIGURE 4:**
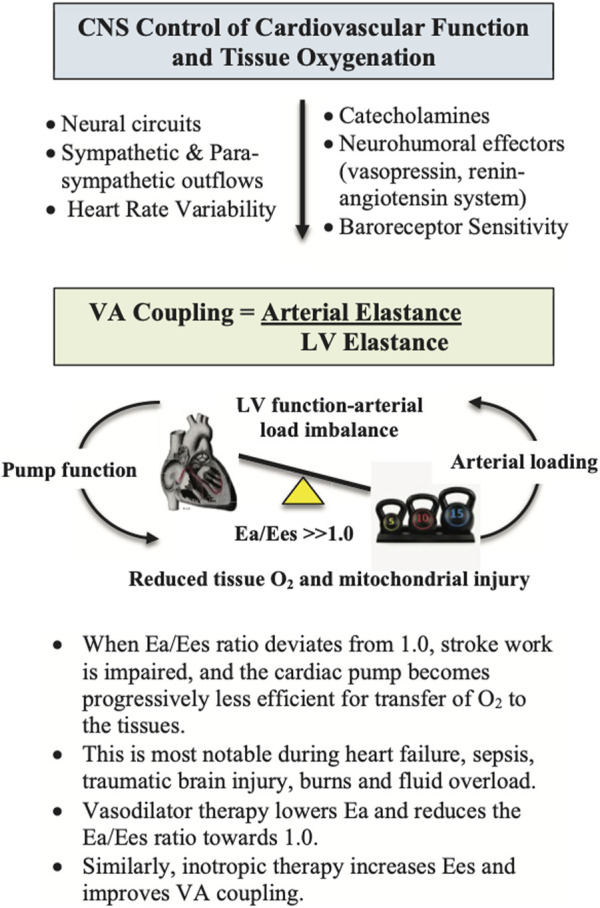
Ventriculo-Arterial (VA) coupling (Ea/Ees) is a measure of mechanical efficiency of heart performance and vascular load function to deliver sufficient O_2_ to the tissues (Monge Garcia et al., 2020). The function of the arterial system is determined by the relationship between the stroke volume (SV) and end-systolic arterial pressure, where higher SVs lead to higher arterial end-systolic pressures (London, 2005; Yurdagul et al., 2016; Axell et al., 2017). The slope of this relationship is termed arterial elastance (Ea), which describes the capability of the arterial vessels to increase pressure when SV increases. Ea is estimated as 0.9 times the brachial systolic pressure divided by SV. Ees is a measure of cardiac contractility and can be determined non-invasively using routine echocardiography (Guarracino et al., 2013). It is a load-independent index of left ventricular (LV) chamber performance, which is influenced by wall stiffness, fibrosis, contraction synchrony and geometric LV chamber dimensions. When Ea/Ees ∼1.0, the efficiency of the system is optimal, meaning that the left ventricle is providing sufficient SV at its lowest possible myocardial energy consumption. The advantage of VA coupling over ejection fraction (EF) or cardiac output (CO) is that it provides additional information on arterial loading and left ventricular function to potentially guide treatments.

It has been estimated that around 30% of polytrauma patients have abnormal ventricular wall motion abnormalities and troponin 1 release with concomitant falls in stroke volume (SV) and cardiac output (CO), despite inotrope and vasopressor support (Wall et al., 2019; Weber et al., 2021). In addition, aggressive fluid replacement, particularly in hypovolemic patients, can further increase the risk of adverse cardiac events and mortality (Wall et al., 2019; Weber et al., 2021). Although smaller resuscitation fluid volumes are recommended (200–500 ml), some patients respond to the fluid challenge with increased SV and CO, while others do not, indicating preload is only one factor in rebalancing cardiac performance (Marik and Lemson, 2014; Ueyama and Kiyonaka, 2017). A fundamental link between central control, cardiovascular function and tissue oxygen supply is ventriculo-arterial (VA) coupling (Figure 4). VA coupling is a concept that developed from the idea that the heart and arterial system are inherently related (Monge Garcia and Santos, 2020). It is the ratio of arterial elastance (Ea) to left-ventricular (LV) elastance (Ees) (Guarracino et al., 2013; Cholley and Le Gall, 2016; Monge Garcia et al., 2020), that can be measured from routine echocardiography (Suga et al., 1993; Kass, 2005; London, 2005; Guarracino et al., 2013; Cholley and Le Gall, 2016; Dobson et al., 2017; Monge Garcia et al., 2020). When the ratio is close to unity, the efficiency of transfer is considered optimal. If the ratio is excessively low or high, the heart as a pump and the vascular load become uncoupled and tissue perfusion is compromised (Guarracino et al., 2013; Onorati et al., 2013; Granfeldt et al., 2014). Uncoupling refers to a disconnect between the pumping action of the heart and the load generated by the arterial system. *The clinical advantage of VA coupling over gold standard ejection fraction (EF) or cardiac output (CO) is that it provides LV function and arterial load properties* (Figure 4) (Monge Garcia and Santos, 2020; Guarracino et al., 2013; Cholley and Le Gall, 2016). For example, if the proximal arteries become stiff, as a result of vasomotor dysregulation, the afterload on the heart increases (Ye et al., 2015), and if the heart becomes stiff it cannot relax optimally to fill and eject sufficient blood into the conduits (Cholley and Le Gall, 2016). Combined they can lead to VA uncoupling, tissue hypoperfusion and multiple organ dysfunction syndrome (MODS) (Antonini-Canterin et al., 2013; Ky et al., 2013). In short, VA coupling reflects the efficiency of the heart to pump blood and the ability of the arterial system to receive it (Monge Garcia and Santos, 2020; Cholley and Le Gall, 2016).

Following major trauma, VA uncoupling not only affects the periphery but it can lead to hypoperfusion of the abdominal organs, including gut wall ischemia and leakiness, which can exacerbate immuno-inflammatory trajectories, immunosuppression, infectious complications and sepsis (Mayer and Gupta, 2015). Alterations in the gut microbiome is bidirectionally linked to the CNS through vagal afferents and HPA axis, and the CNS to the GI tract via the enteric nervous system (Tillisch, 2014; Mayer and Gupta, 2015; Liang and FitzGerald, 2017; Letson et al., 2019a; Dobson et al., 2019; Morris et al., 2019). In trauma patients, Howard and colleagues reported rapid changes in the microbiome following injury, which were associated with poor outcomes (Howard et al., 2017). However, further clinical studies are required to understand the role of the gut-brain axis, VA uncoupling and poor outcomes after major trauma.

## Second pillar: The endothelial glycocalyx

Along the outer surface of the endothelial cell is a fluffy density which represents a portion of the glycocalyx of this cell, and which is usually called the capillary basement membrane ... It is made up of a tangle of fine filaments less than 100 A (10 nm) in diameter … I hope my speculations and suggestions may stimulate new research and new experiments.

Bennet H.S. (Bennett, 1963) p17

The endothelial glycocalyx is a key player to VA coupling through the exchange of material between the blood and tissues (Figure 3). Material exchange includes O_2_, metabolic fuels, hormones, immune cells, immuno-inflammatory regulators and fluids (Aird, 2005; Wiel et al., 2005; Rahbar et al., 2015; Huang and Godula, 2016; Johansson et al., 2017a; Gonzalez Rodriguez et al., 2017; Halbgebauer et al., 2018; Richards et al., 2021). Under normal conditions, the luminal glycocalyx ‘fuzz’ also acts as a highly regulated vascular filter overlying the endothelial cell-cell junctions, which contains an estimated 1 to 1.7 L of non-circulating plasma (Schott et al., 2016; Hahn, 2020; Moore et al., 2021a). Traditionally, the vascular endothelium is believed to cover a surface area (SA) of 3,000–7,000 m^2^ (Figure 3) (Aird, 2005; van Hinsbergh, 2012). We have recently revised this estimate to include the glycocalyx “fuzz”, and found the SA increases by more than ten-fold to over 46,000 m^2^ or over ∼200 tennis courts or 8 United States football fields (Figure 3) (Dobson et al., 2022b). This provides a new perspective of the enormous substrate and susceptibility of the body for inflammation, coagulopathy and mitochondrial exchange in health and disease.

Following major trauma, damage to endothelial-glycocalyx occurs almost immediately at the point of injury, and is termed Endotheliopathy of Trauma (EoT) (Naumann et al., 2018). EoT is characterized by endothelial activation, vasoactivity, fluid shifts, leakiness, leukocyte adhesion, inflammation, coagulopathy and mitochondrial dysfunction (Tiruppathi et al., 2003; Reitsma et al., 2007; Chappell et al., 2009; Biddle, 2013; Aditianingsih and George, 2014; Gall et al., 2019; Moore et al., 2021b). Endothelial activation is triggered by changes in blood flow and shear stress, local injury and circulating immune cells and DAMPs, that can lead to shedding of the glycocalyx, which is normally anchored to underlying cells (Lipowsky, 2012; Fu and Tarbell, 2013; Jin et al., 2021). Shedding is mediated by enzymatic membrane-bound ‘sheddases’, which are activated by reactive oxygen species and other signals in response to the traumatic stress (Lipowsky, 2012; Jin et al., 2021).

Upon activation and shedding, endothelial cells release nanoscale bioactives, such as thrombomodulin, tight junction proteins, syndecan-1, heparan sulphate, hyaluronic acid, and other proteoglycans and glycoproteins, into the circulation (Bennett, 1963; Luft, 1966; Bazzoni and Dejana, 2004; Reitsma et al., 2007; Woodcock and Woodcock, 2012; Aditianingsih and George, 2014; Chappell and Jacob, 2014). After traumatic injury, these injury markers indicate widespread tissue damage, including damage to the blood brain barrier (BBB) (Greene et al., 2019; Jin et al., 2021). Disruption to the BBB is important because the brain loses its ‘immune privilege’ over the rest of the body (Figure 2). However, if tissue perfusion is restored early, the glycocalyx has a remarkable capacity to repair itself and restore its barrier functions (Zeng and Tarbell, 2014; Banerjee et al., 2021; Jin et al., 2021). Timing of repair appears to depend upon the duration and extent of hypoperfusion, and the type and severity of trauma. Naumann and colleagues recently reported, for example, that microvascular impairment in 19 trauma patients was still prominent ∼10 h post-injury (Naumann et al., 2018). Protecting and restoring the glycocalyx is a potential target for new drug therapies (Torres Filho et al., 2017; Banerjee et al., 2021).

## Third pillar: Mitochondrial integrity and organ dysfunction

Mitochondria are central hubs for sensing certain types of mild to moderate stress, and signal to initiate appropriate cellular responses.

Berry and colleagues (Berry et al., 2018) p9

The third pillar of SHOT is regulation of the system’s energy requirements (Figure 3). Mitochondria are terminal structures of the respiratory system where the potential energy from the food we eat is converted to ATP at the expense of O_2_ utilization (Dobson, 2003; West et al., 2011). These powerhouses are of ancient bacterial origins and involved in substrate regulation, cell signalling, calcium homeostasis, endoplasmic reticulum communication and cell death regulation (Kluge et al., 2013; Bhatti et al., 2017; Aswani et al., 2018; Berry et al., 2018; Hauser and Otterbein, 2018; Thurairajah et al., 2018). After severe trauma and bleeding, ATP can no longer be replenished fully by mitochondria and the tissues transition to a blend of oxidative phosphorylation and anaerobic metabolism (Figure 3). However, this strategy is an emergency system that can only be sustained for short periods of time, after which tissue dysfunction and organ failure occur. Injured and dying cells release their mitochondrial DAMPs into the circulation, which further stresses the CNS system, discussed above (Figure 2) (Boudreau et al., 2014; Cap and Hunt, 2014; Zhao et al., 2016; Aswani et al., 2018). Like mitochondria, there is significant evolutionary conservation between mitochondrial DAMPs (and PAMPs), that helps to explain the parallels between endotoxic shock and traumatic shock (Zhang et al., 2010). A number of therapeutic strategies have been proposed to scavenge these DAMPs and break the secondary injury cycle (Aswani et al., 2018; Thurairajah et al., 2018). However, most studies have had limited success.

## Other systems-based, unifying models of trauma

Shock-induced endotheliopathy (SHINE) is observed in acute critical illness and may reflect a potential unifying pathophysiologic mechanism linked to poor outcome. Sympatho-adrenal hyperactivation appears to be a pivotal driver of this condition.

Johansson and colleagues (Johansson et al., 2017b) p5

In 2017, Johansson and colleagues introduced a model of shock-induced endotheliopathy (SHINE) to understand the underlying mechanisms for critically ill patients (Johansson et al., 2017b). They proposed that shock-induced sympatho-adrenal hyperactivation was a critical driver of tissue hypoperfusion, endothelial damage, and subsequent hemostatic aberrations and MODS. More recently, the same group found that patients suffering from the same trauma severity were highly heterogenous in their endothelial responses, as measured by syndecan-1, and proposed a minimum of four shock-induced endotheliopathy phenotypes (Henriksen et al., 2020). They concluded that the endothelial responses were highly heterogeneous, and most likely driven by a genetic component (Henriksen et al., 2020). A difference between SHOT and SHINE is the functional linkage between CNS, VA coupling and the endothelial glycocalyx (Figure 4). Without maintaining a tight VA coupling to deliver sufficient oxygen to tissue mitochondria, SHOT proposes that the vascular endothelium and BBB will continue to be activated and secondary injury perpetuated (discussed above).

## Tentative explanations for early and late traumatic deaths

The tragedies of life are largely arterial.

Sir William Osler (1908) Quoted from Criado (Criado, 2011)

**FIGURE 5:**
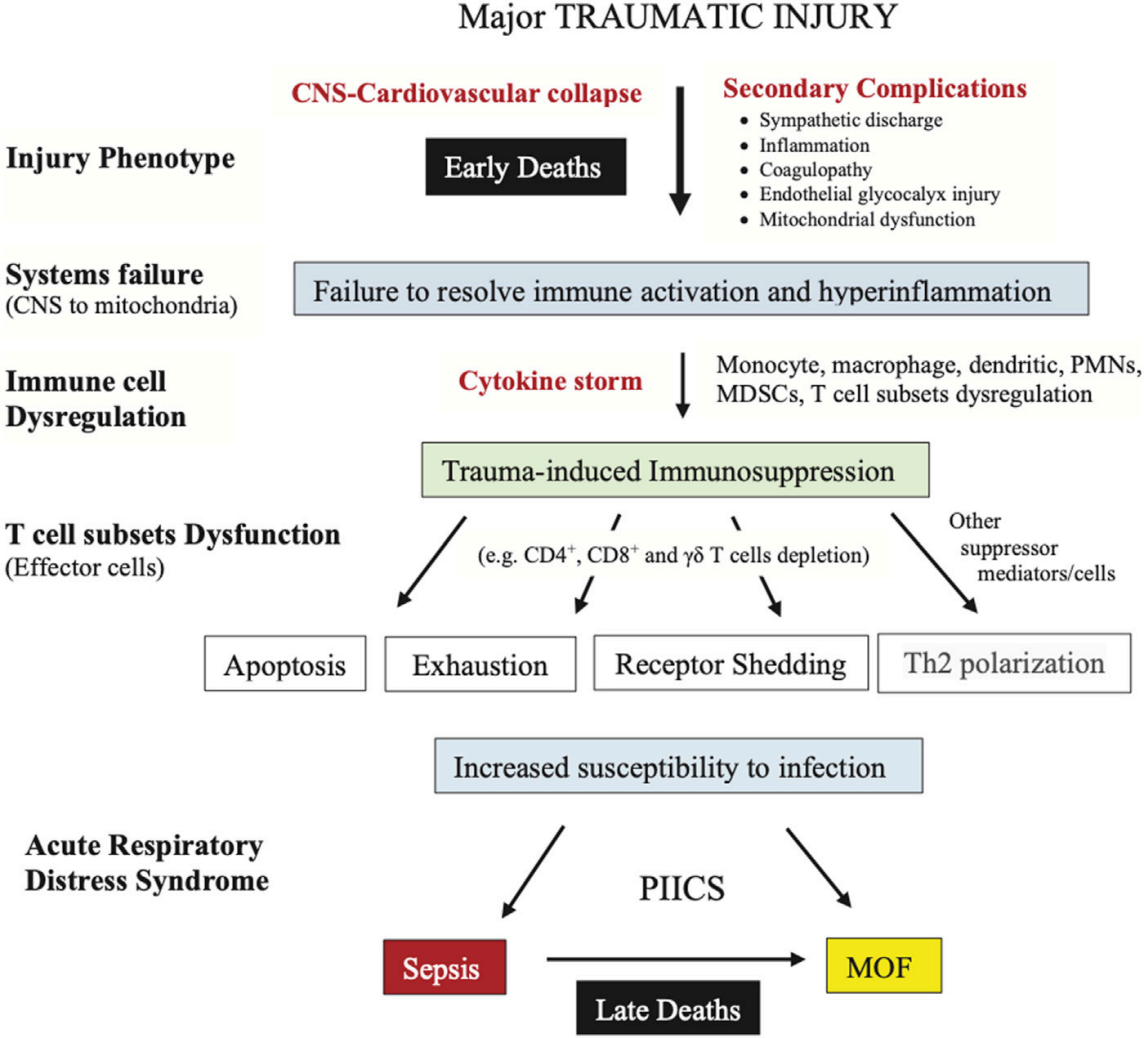
Schematic of trauma-induced immunosuppression involving different dysfunctional states of immune T cells that can increase the host’s susceptibility to infection, sepsis and late death. Some of the cellular suppressors include the myeloid progenitor cells, immature macrophages, immature granulocytes and immature dendritic cells. If immune dysregulation and inflammation is left unchecked, it can lead to physiological exhaust, respiratory failure, Persistent Inflammation, Immunosuppression and Catabolism Syndrome (PIICS) and multiple organ failure (MOF). MDSC, myeloid-derived suppressor cell; PMN, polymorphonuclear leukocyte.

Having presented a systems hypothesis of trauma, we now return to the question posed by Brohi and colleagues: “Why are certain groups of severely bleeding trauma patients still dying? (Figure 1) (Brohi et al., 2019). We suggest the first wave of mortality is due to early failure of the CNS-cardiovascular system to maintain VA coupling close to or around unity with subsequent loss of tissue oxygenation (Figures 3, 4). Extreme uncoupling of flow to arterial load would rapidly lead to physiological exhaust from widespread hypoperfusion, glycocalyx shedding, mitochondrial ATP deficit, sympathetic dominance, cardiovascular failure, unchecked coagulopathy, uncontrolled inflammation and multiple organ failure (Figure 2). In the second wave, we suggest that a failing CNS-cardiovascular system is still present, however, not to the same extent as the early mortality group. In delayed deaths, secondary injury will continue to progress until the body’s immune defence is exhausted and the system fails from multiple organ dysfunction (Figure 5).

Understanding the mechanisms of immunosuppression and organ dysfunction have been a challenge (Islam et al., 2016; Cabrera et al., 2017; Roth et al., 2021; Dobson et al., 2022b). In a landmark study, Mansen and colleagues examined early changes in circulating lymphocytes and showed that trauma patients who developed MODS within 24 h had nearly 2-fold higher CD56^dim^ NK cells, 80% lower gamma delta (γδ)-low T cells and 4-fold higher interferon (IFN)-γ upon hospital admission, compared to patients who did not (Manson et al., 2016). Moreover, they showed that patients who developed MODS also developed lymphopenia within 24 h of injury, which if persisted to 48 h led to high mortality (Manson et al., 2016). Poor outcomes may further be aggravated from progressive gut ischemia (Morris et al., 2019; Yuan et al., 2021). The authors propose that early events may be pivotal to the development of a “normal” or “dysregulated” immune response (Manson et al., 2016; Cabrera et al., 2017). Other determinants of late death include type and severity of trauma, retrieval time, age, previous health status, socioeconomic status, sex and other genetic factors.

**FIGURE 6:**
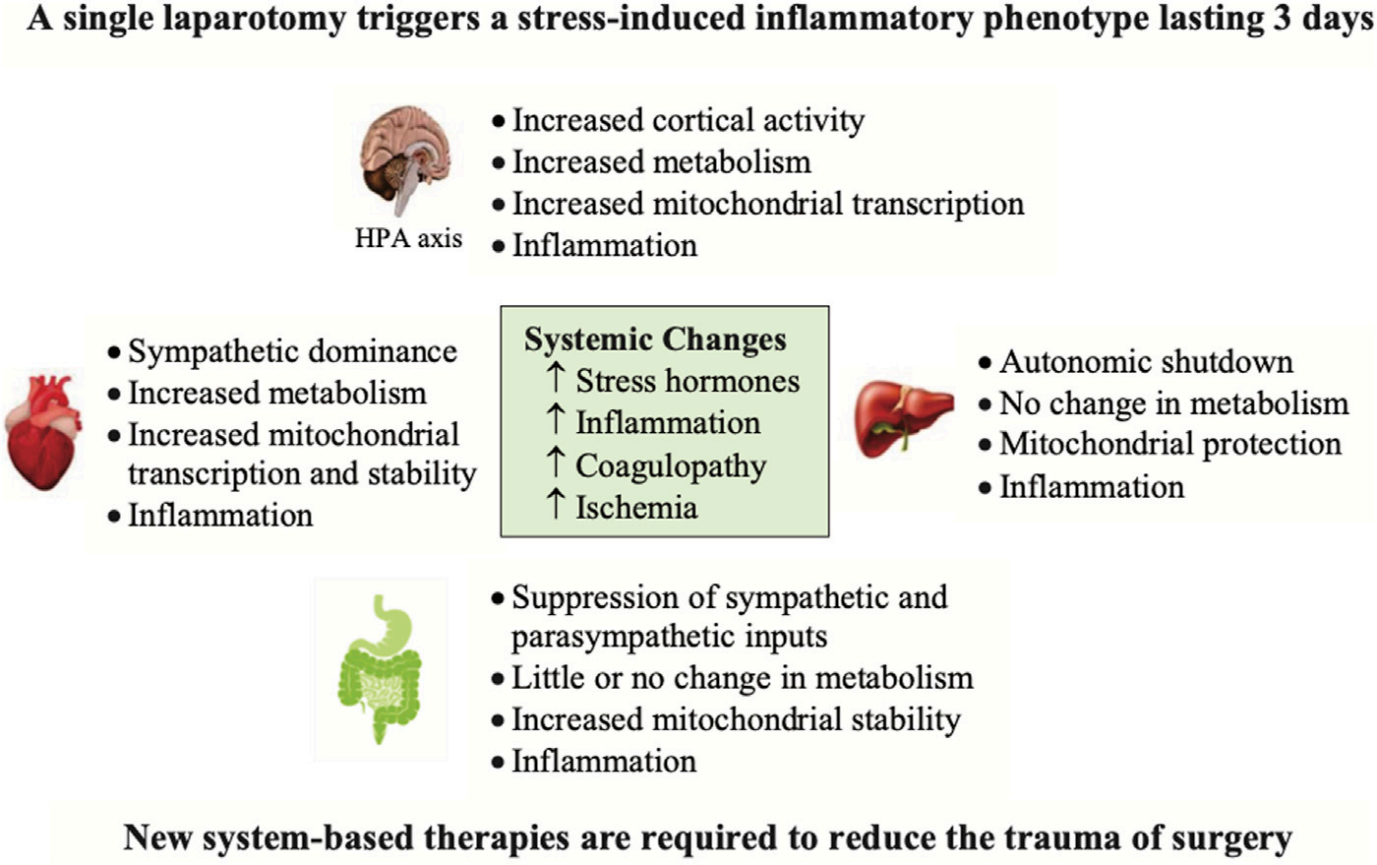
The effect of a single transverse laparotomy on receptor gene expression in a number of tissues of the rat over a 3 day period (Dobson et al., 2021a). The trauma of surgery led to a proinflammatory phenotype involving neuroendocrine stress, cortical excitability, immune activation, lymphocytopenia, hypermetabolism and coagulopathy (Dobson et al., 2021a). Of special note was the profound hyperactivity of the brain and heart with no measurable changes in hemodynamics. In contrast, the liver and gut underwent downregulation of adrenergic and muscarinic receptor expression. This study illustrates the widespread effect of a single incision mimicking an abdominal trauma on altering brain and whole body homeostasis with no further surgery or manipulation (Dobson, 2020a; Dobson et al., 2021a). HPA, hypothalamo-pituitary axis.

Another key contributor to early and late deaths is the patient’s response to surgery itself. As discussed above, the trauma of surgery may reduce the patient’s physiological reserve and exacerbate immune dysregulation, inflammation, coagulopathy and multiple organ dysfunction (Dobson, 2020a; Dobson et al., 2021a). We recently showed that a single laparotomy, with no further surgery, induced an immune-triggered proinflammatory phenotype involving neuroendocrine stress, cortical excitability, immune activation, hypermetabolism and coagulopathy (Figure 6) (Dobson et al., 2021a). Accompanying the trauma, were significant increases in M1 muscarinic (31-fold) and α-1A-adrenergic (39-fold) receptor expression in brain cortex and 6-fold increases in proinflammatory cytokine interleukin (IL)-1β expression over 3 days (Dobson et al., 2021a). These early and persistent changes in the anesthetized brain after surgery support Crile’s proposal that it is still ‘wide awake’ to receive DAMPs, and other signal stressors, originating from the first incision and subsequent secondary effects (Crile, 1913).

Similarly, in heart there were 8-fold increases in β-1-adrenergic receptor expression, and up to 6-fold increases in expression of M2 and M1 muscarinic receptors after 6 h despite no changes in hemodynamics (Figure 6). Lymphocyte levels also decreased by ∼70% at 6 h and 3 days, and IL-10 dramatically increased from undetectable baseline levels to 483 pg/ml after 6 h with further increases at 3 days (1,149 pg/ml) (Dobson et al., 2021a). Blood lactate also increased indicating that aerobic glucose metabolism was insufficient and required a ‘boost’ from anaerobic pathways to replenish ATP (Dobson et al., 2021a). These ‘silent’ changes in brain and heart are remarkable given there was only one abdominal incision with no further injury to these organs (Figure 6). It would be interesting to repeat the study with a local anesthesic administered to the incision line prior to the laparotomy to determine if cortical excitability, and subsequent downstream effects were reduced.

## The path forward: Towards an adenosine, lidocaine and magnesium ‘switch’ hypothesis

The challenge for the future is to develop new ‘upstream’ drug therapies that target the CNS stress response and hemorrhage control as close to the point-of-injury as possible. *The therapeutic goal is to ‘switch’ the genomic and proteomic networks from an injury phenotype to a survival phenotype.* The term ‘switch’ is not like a light switch but a ‘transitional’ phenomic switch involving multiple mechanisms. Currently, no effective drug therapy exists that targets the system. For over a decade, we have been developing a drug comprising adenosine, lidocaine and magnesium (ALM) for major trauma and surgery (Dobson and Letson, 2016; Dobson, 2020a; Dobson and Letson, 2020). Preclinical studies show some promise in shifting sympathetic to parasympathetic dominance, maintaining VA coupling ratio close to one, reducing non-compressible bleeding, correcting coagulopathy, suppressing immune dysfunction, blunting inflammation and lowering energy demand (Granfeldt et al., 2014; Letson and Dobson, 2015; Dobson and Letson, 2016; Letson and Dobson, 2017a; Letson and Dobson, 2017b; Letson et al., 2019b; Dobson and Letson, 2020; Dobson et al., 2021a; Letson et al., 2022).

ALM fluid therapy appears to support a high-flow, hypotensive, vasodilatory state with maintained endothelial-glycocalyx patency and mitochondrial function (Granfeldt et al., 2015; Letson et al., 2020). In addition, Dubick and colleagues from the US Army Institute of Surgical Research independently reported that ALM therapy nearly completely reversed endothelial glycocalyx damage after severe hemorrhagic shock (Torres Filho et al., 2017; Banerjee et al., 2021), which is consistent with our findings of rapid 5 min correction of coagulopathy following different traumatic injuries (Letson et al., 2012; Letson and Dobson, 2015; Letson and Dobson, 2018; Dobson et al., 2022b; Letson et al., 2022). A curious result of ALM therapy is that it confers multi-protection against: 1) sterile injury (Dobson and Letson, 2016; Dobson and Letson, 2020; Dobson et al., 2021b), 2) infection (Griffin et al., 2014; Griffin et al., 2016) and 3) lipopolysaccharide (LPS)-induced endotoxemia (Granfeldt et al., 2013), which implies a common mechanism of action to blunt DAMPs, PAMPs and other inflammatory signals.

**FIGURE 7:**
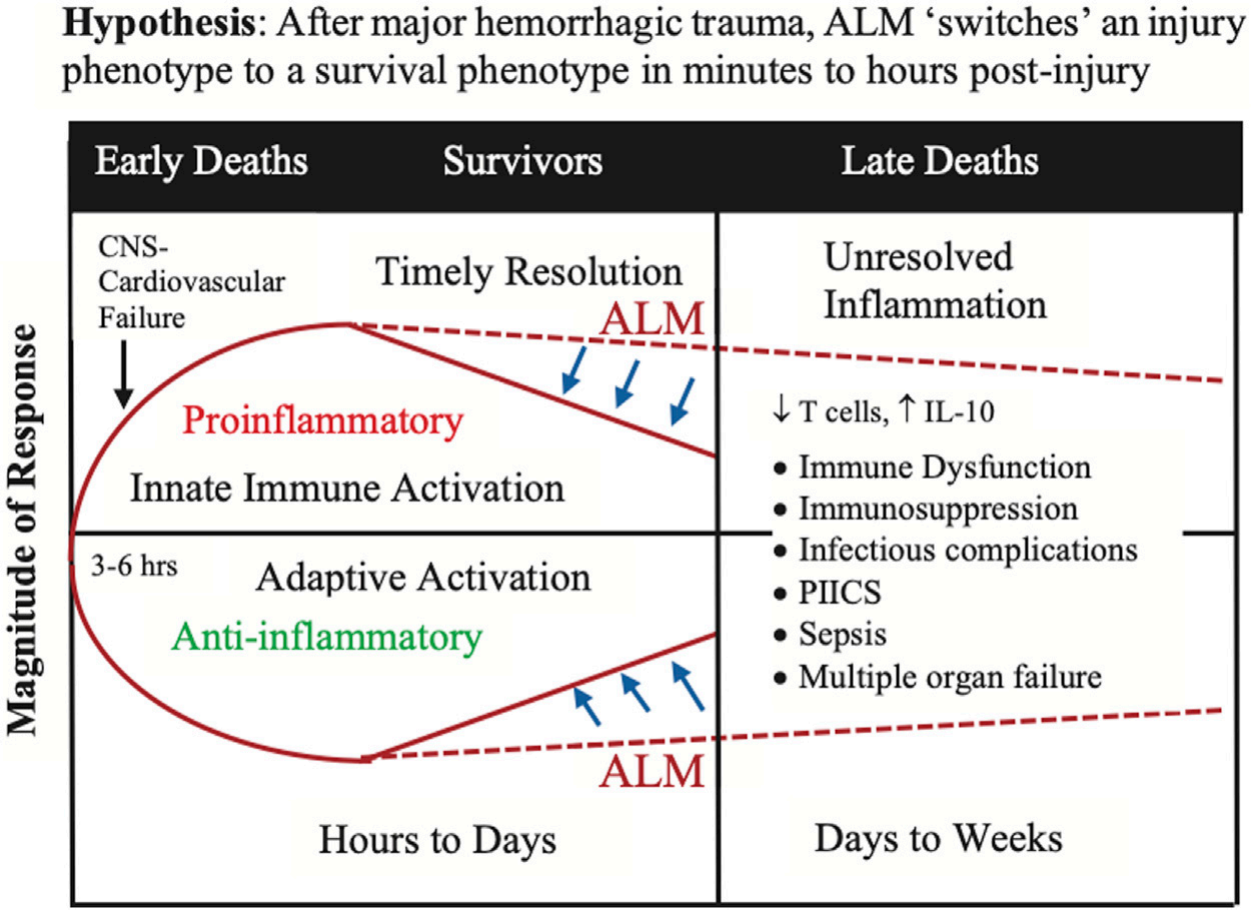
The ‘switch’ hypothesis proposes that ALM therapy transitions the injury phenotype to a survival phenotype in the first few minutes to hours after major trauma. The switch involves re-setting or rebalancing the innate immune and inflammatory responses to the surge and ongoing releases of DAMPs, and other damage signals, into the circulation from the trauma. Implicit to the hypothesis is the assumption that the hyperacute immune and inflammatory events that occur in first few minutes to hours following a major trauma pre-determine the trajectory of the later immune and inflammatory responses and outcome. Timely resolution of the immune and inflammatory ‘storms’ appears to be key. Although the mechanisms are unknown, one potential target are monocytes which have recently been shown to sense injury-released DAMPs via the AIM2 inflammasome and induce the extrinsic cell death of T cells (Roth et al., 2021). ALM, adenosine, lidocaine and magnesium; CNS, central nervous system; DAMP, damage-associated molecular pattern; IL, interleukin; PIICS; Persistent Inflammation, Immunosuppression and Catabolism Syndrome.

Although we don’t know when or how the phenomic ‘switch’ occurs, it is possible ALM acts in the first minutes to hours post-injury to blunt the CNS sympathetic outflows, as part of the stress response to trauma, and protect the blood brain barrier (BBB) that prevents circulating immune cells, DAMPs and proinflammatory mediators from entering the brain. Protecting the CNS and BBB may maintain the brain’s ‘immune privilege’ status over the rest of the body, and possibly reduce the innate immune and inflammatory genomic ‘storms’ (Figure 7). *Early treatment with ALM may therefore lead to short-term benefits with long-term outcomes by rebalancing the system with timely resolution of immune dysregulation and systemic inflammation* (Figure 7). Further work is required to test this hypothesis in clinically relevant animal models and translate the therapy to human trauma and surgery, while appreciating that the success rate of translating new drugs to humans is around 5% or less (Downing et al., 2017; Seyhan, 2019). Understanding the survival mechanisms of ALM therapy is essential for safe translation.

## Conclusion

We view the early and late deaths after traumatic hemorrhage as systems failures, not as a series of single-event manifestations that occur over time. We hypothesize that breaks in the system occur early with the flooding of DAMPs and other immune and inflammatory modulators into the circulation and the brain losing its ‘privilege’ status over the rest of the body. Another contributor to early and late deaths is from the trauma of emergent surgery itself, which adds a further stress to central control that perpetuates immune dysregulation, inflammation, immunosuppression, infection and MODS. We have been developing a new ALM point-of-care drug therapy for prehospital trauma and to reduce the trauma of major surgery, that could be administered immediately after anesthesia and before the first incision. Finally, greater emphasis should be placed on ‘the system’ in civilian and military medicine, and in teaching, medical training and drug development programs.
